# Sparse self-attention aggregation networks for neural sequence slice interpolation

**DOI:** 10.1186/s13040-021-00236-z

**Published:** 2021-02-01

**Authors:** Zejin Wang, Jing Liu, Xi Chen, Guoqing Li, Hua Han

**Affiliations:** 1 National Laboratory of Pattern Recognition, Institute of Automation, Chinese Academy of Sciences, 95 Zhongguancun East Road, Beijing, 100190 China; 2 School of Artificial Intelligence, University of Chinese Academy of Sciences, 19 Yuquan Road, Beijing, 100190 China; 3 Center for Excellence in Brain Science and Intelligence Technology Shanghai Institutes for Biological Sciences, Chinese Academy of Sciences, 320 Yue Yang Road, Shanghai, 200031 China; 4 School of Future Technology, University of Chinese Academy of Sciences, 19 Yuquan Road, Beijing, 100190 China

**Keywords:** Slice interpolation, Biological tissue recovery, EM images, Sparse self-attention network, Adaptive style-balance loss

## Abstract

**Background:**

Microscopic imaging is a crucial technology for visualizing neural and tissue structures. Large-area defects inevitably occur during the imaging process of electron microscope (EM) serial slices, which lead to reduced registration and semantic segmentation, and affect the accuracy of 3D reconstruction. The continuity of biological tissue among serial EM images makes it possible to recover missing tissues utilizing inter-slice interpolation. However, large deformation, noise, and blur among EM images remain the task challenging. Existing flow-based and kernel-based methods have to perform frame interpolation on images with little noise and low blur. They also cannot effectively deal with large deformations on EM images.

**Results:**

In this paper, we propose a sparse self-attention aggregation network to synthesize pixels following the continuity of biological tissue. First, we develop an attention-aware layer for consecutive EM images interpolation that implicitly adopts global perceptual deformation. Second, we present an adaptive style-balance loss taking the style differences of serial EM images such as blur and noise into consideration. Guided by the attention-aware module, adaptively synthesizing each pixel aggregated from the global domain further improves the performance of pixel synthesis. Quantitative and qualitative experiments show that the proposed method is superior to the state-of-the-art approaches.

**Conclusions:**

The proposed method can be considered as an effective strategy to model the relationship between each pixel and other pixels from the global domain. This approach improves the algorithm’s robustness to noise and large deformation, and can accurately predict the effective information of the missing region, which will greatly promote the data analysis of neurobiological research.

## Background

Inter-slice interpolation is important in electron microscope (EM) image analysis. The destruction of the biological tissues during sample preparation and EM imaging can cause large-area defects in serial EM images. Recent methods based on context information are effective for small area defects, but cannot handle large area. The continuity of the biological tissue of the serial slices contributes to predicting the missing information despite the failure of using only the spatial information of a single EM image. To date, there are only few reported works available in the field of sequence slice interpolation [1–3], and the EM image restoration methods are not effective when dealing with large-area defects. However, interpolation methods can accurately predict non-defective intermediate frames, which can replace original intermediate frames with large-area defects. Besides, non-defective intermediate images contribute to improve registration with sudden and significant structural changes, improve semantic segmentation accuracy [4], and ensure 3D reconstruction continuity [5].

For optical images, with the development of deep learning, frame interpolation has gone through five stages: simple CNN-based methods [6], deep voxel flow-based methods [7], kernel-based methods [8, 9], motion-based methods [10, 11] and depth-based methods [12]. Long et al. [6] first adopted a generic CNN-based network synthesizing the intermediate frame directly. However, the results suffer from severe blurriness since generic CNN cannot obtain the multi-modal distribution of optical images and videos. Then, Liu et al. [7] proposed the deep voxel flow to warp input frames based on a trilinear sampling. Although the intermediate frames generated from voxel flow suffer low blurriness, the procedure of flow estimation remains a challenge for large motion.

Instead of adopting optical flow to handle significant motion, Niklaus et al. [8, 9] proposed a spatially-adaptive interpolation kernel to synthesize pixels from a large neighborhood. However, these kernel-based methods only build dependencies from local areas and typically require heavy computation cost when the size of kernel increases. Then, Bao et al. [11] integrate kernel-based and flow-based approaches into an end-to-end network to benefit from both sides. Recently, Bao et al. [12] further introduced depth estimation to the previous work, which explicitly deals with occlusion. Existing flow-based methods utilize kernel estimation to improve the precision and robustness of single-pixel synthesis. However, pixels synthesized by kernel estimation only consider local neighborhood information. In general, existing interpolation methods deal with occlusion, significant motion, adopting depth maps [13–15], optical flow, and local interpolation kernels. However, on EM images with large deformation, drift, and abundant noise, estimation of the accurate optical flow field [16–18] suitable for sequence EM images remains a challenge. Furthermore, the ultimate goal is to synthesize high-quality intermediate frames without defect, and optical flow estimation is used only as an intermediate step. Kernel-based methods [8, 9] behave well owing to combine flow estimation and pixel synthesis into a single step. The kernel estimation synthesizes the intermediate frame pixels through the spatial kernel based on the traditional convolution. However, it cannot establish the dependence of the global domain, and when the spatial kernel is expanded to the input image size, the computation and memory complexity is not lower than the original self-attention mechanism.

Focusing on finding more accurate deformation fields on EM images, early researchers also proposed a series of traditional slice interpolation methods, including shape-based methods [19], morphology-based methods [20], registration-based methods [21]. However, the implementation of these conventional methods was based on an essential assumption that changes in the structure must be sufficiently small. This assumption makes the methods mentioned above unsuitable for sparsely sampled slices. Recently, with the development of deep learning [22–24], there are several CNN-based slice interpolation methods. [1] proposed a simple convolutional auto-encoder for binary image interpolation. Then, Nguyen et al. [3] leveraged slice interpolation to improve registration. Slice interpolation was only an auxiliary part of registration. Wu et al. [2] proposed an Intermediate slice synthesis model for boosting medical image segmentation accuracy. The slice synthesis model was based on the kernel estimation method [9] and cannot handle noise and blur differences, large deformation, and drift among EM images.

Recently, the self-attention mechanism has become an integral part of the entire model, to establish global dependency for each position. Self-attention, also called intra-attention, was originally proposed to calculate the response at a position in a sequence, then it was first plugged into machine translation [25], achieving state-of-the-art results. Parmar et al. [26] proposed an Image Transformer model adopting self-attention for image generation. Wang et al. [27] proposed non-local operations to model the spatial-temporal dependencies in various computer vision tasks, e.g. video classification, object detection, and instance segmentation. Recently, some researchers [28–30] applied a similar mechanism for semantic segmentation and achieved good performance.

Despite its progress, self-attention has not been applied in neural sequence slice interpolation. Inspired by the works above, we propose a simple and efficient multi-level sparse strategy to decompose the original affinity matrix of the self-attention mechanism into the product of two sparse affinity sub-matrices, and we apply the interlacing mechanism to group the pixels with long spatial interval distances together for the long-range attention. If the size of the sparse affinity sub-matrix is larger than the threshold, the sub-matrix continues to decompose itself in the same way. Notably, the concurrent works, Sparse Transformer [31] and Interlaced Sparse Self-Attention [30] also adopt similar factorization scheme to improve the efficiency of self-attention on sequential tasks and semantic segmentation while we focus on consecutive EM image interpolation. In contrast, we implicitly detect global deformation and integrate pixels from global dependency by utilizing the self-attention information in the attention-aware layer. Furthermore, We utilize the multi-level sparse strategy further to improve the computational efficiency of the self-attention mechanism. Moreover, we replace traditional kernel estimation with the proposed attention-aware layer to synthesize pixels from global dependency.

To address the problems above, we introduce a simple and efficient solution named attention-aware layer (AAL). The AAL perceives all positions in the input frames, and then synthesize each pixel of the intermediate frame according to the attention maps. In more detail, AAL learns to focus on global deformation without additional supervision, implicitly considering all positions in the input frames to generate each pixel in the middle frame. In this way, the optical flow field extraction and kernel estimation can be reasonably removed while maintaining the intermediate frame accuracy. Besides, a two-level sparse self-attention mechanism in AAL decreases the computation and memory complexity substantially. Considering the style differences in the degree of noise and blur between the serial EM images, we also propose an adaptive style-balance loss, which strengthens the supervision of input frames and ensure the natural transition of three consecutive frames. As a result, our proposed approach performs better than other methods on EM images.

In this paper, we propose a novel AAL that can perfectly replace the kernel estimation layer and establish a global dependence for each pixel. Additionally, we explore the effects of different loss functions on the EM image interpolation task. In particular, we propose an adaptive style balance loss. The main contributions can be roughly grouped in three different directions.

- We present an attention-aware layer to capture the dense long-range dependencies for each pixel with lower memory and computation consumption. The proposed module improves performance compared to kernel-based methods and flow-based methods. Moreover, our approach combines flow estimation and kernel estimation into a single step
- We propose the style balance loss to handle differences in style among three input consecutive EM images. The proposed loss not only guides the style of generating intermediate frames to be closer to the ground truth but also utilizes the styles of the front and rear frames to strengthen the constraints on the intermediate frame style. We show that using front and rear frame styles for supervision can better generate intermediate frames with natural transition.
- Based on CREMI^1^ dataset, provided by MICCAI 2016 Challenge as serial section transmission electron microscopy (ssTEM) images, we generate a new dataset, named *cremi*_*triplet*, for the task EM image interpolation. Besides, we also generate a dataset named *mouse*_*triplet* for interpolation based on automatic tape-collecting ultramicrotome (ATUM) mouse brain data. Experimental results demonstrate the effectiveness of the attention aware layer on EM image interpolation, which is superior to the kernel-based methods and flow-based methods.

## Materials and methods

**Fig. 1:**
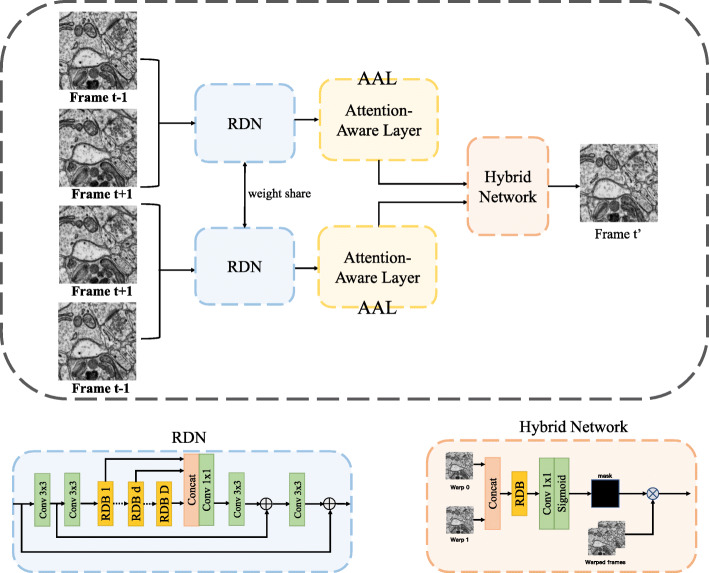
Overview of the SSAN algorithm, which includes the siamese residual dense network, attention-aware layers, and hybrid network. Given two input EM images, we first use the RDN module to calculate the forward and reverse features and then use the proposed attention-aware layer to generate warped intermediate frames. We then use a hybrid network adaptively fusing the warped intermediate frames to generate the final intermediate frame

In this paper, we propose a sparse self-attention aggregation network (SSAN) for EM image interpolation. An overview of the proposed attention-aware interpolation algorithm is shown in Fig. 1, which is primarily based on the siamese residual dense network, attention-aware layer, and hybrid network. Given two input frames **I**_*t*−1_ and **I**_*t*+1_, the goal is to synthesize an intermediate frame $\hat {\mathbf {I}}_{t}$. We first encode the feature maps, denoted by **F**_*t*−1→*t*+1_ and **F**_*t*+1→*t*−1_, through siamese residual dense network. Then, the proposed attention-aware layer synthesizes the warped frames **warp**_0_ and **warp**_1_ based on **F**_*t*−1→*t*+1_ and **F**_*t*+1→*t*−1_. After obtaining the warped frames, the proposed hybrid network generates the interpolated frame $\hat {\mathbf {I}}_{t}$ by element-wise linearly fusing.

### Dataset and preprocessing

Note that there is no public dataset for the EM image interpolation task, we generate the ground truth of two major types of EM images: ssTEM images and ATUM images. To be specific, we use the ssTEM images from the CREMI dataset provided by MICCAI 2016 Challenge on https://cremi.org/, and the ATUM images generated from our home grown mouse brain dataset. The CREMI dataset consists of three datasets, each consisting of two 5um^3^ volumes (training and testing, each 1250pixel×1250pixel×125pixel) of serial section EM of the adult fly brain. Each volume has neuron and synapse labelings and annotations for pre- and post-synaptic partners. Taking CREMI’s padder version A dataset as an example, we first convert the hdf5 format A dataset into a png format to obtain 200 images with a resolution of 3072×3072. After that, we utilize the template matching algorithm to align the three consecutive images. Then we traverse from left to right from top to bottom with the stride of 512 and crop the three consecutive images with the resolution of 512×512 after alignment, and save as a sample. Finally, samples with defects, weak continuity and substantial differences in blurring are all deleted. To reduce the difference in brightness and contrast between three consecutive images in each sample, we perform histogram specification operations on both two datasets. The processed CREMI dataset and mouse brain dataset are named as *cremi*_*triplet* and *mouse*_*triplet*, respectively. Each dataset adopts a triplet as a sample for training, where each triplet contains three consecutive EM images with a resolution of 512×512 pixels. There are 3,652 triplets, 2,631 triplets, 1,333 triplets and 2674 triplets in the *cremi*_*triplet* A, *cremi*_*triplet* B, *cremi*_*triplet* C and *mouse*_*triplet*, respectively. Each dataset is divided into a training set, validation set and test set in a ratio of 3:1:1.

### Siamese residual dense network

For feature extractor, the pooling process in the U-Net can damage context information, making it difficult for intermediate frame synthesis. We utilize the residual dense network [32] as the basic feature extractor to preserve the structured information when generating the corresponding hierarchical features of the input frames. As shown in Fig. 1, residual dense network (RDN) mainly consists of three parts: shallow feature extraction net (SFENet), residual dense blocks (RDBs), and finally dense feature fusion (DFF). Besides, the frame interpolation task requires two consecutive frames as input to generate intermediate frames. Here, the siamese structure is adopted, as illustrated in Fig. 1, which preserves the temporal information between consecutive frames during generating hierarchical features and contributes to decreasing the computational consumption.

### Attention-aware layer

**Fig. 2:**
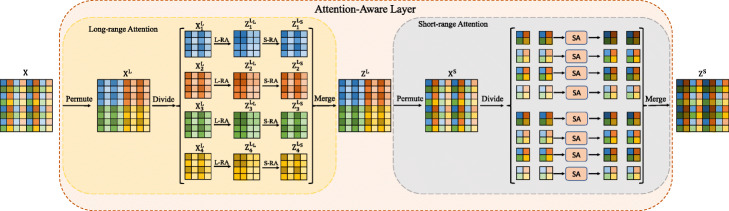
Illustration of Attention-Aware layer. L-RA and S-RA denote long-range attention and short-range attention, respectively. In this figure, we only perform two-level decomposition

After extracting hierarchical features through the siamese residual dense network, the proposed AAL based on multi-level sparse self-attention replaces the kernel estimation. The key of the proposed multi-level sparse self-attention lies in the multi-level decomposition of the original dense affinity matrix **A**, each time decomposing the dense affinity matrix **A** into the product of two sparse block affinity matrices **A**^*L*^ and **A**^*S*^. By combining multi-level decomposition, long-range attention, and short-range attention, pixels of each position can be synthesized from the information of all input positions. We demonstrate how to estimate the long-range attention matrix **A**^*L*^ or the short-range attention matrix **A**^*S*^ and perform multi-level decomposition in Fig. 2.

#### Self-attention

The self-attention [33] scheme is described as below,

1 $$ \mathbf{A}=softmax\left(\frac{(\mathbf{W}_{{f}} \mathbf{X})^{\top}(\mathbf{W}_{{g}} \mathbf{X})}{\sqrt{d}}\right),  $$

2 $$ \mathbf{Z}=\mathbf{W}_{{v}}[\left(\mathbf{W}_{{h}} \mathbf{X}\right)\mathbf{A}],  $$

In the above formulation, $\mathbf {X}\in {\mathbb {R}^{C \times N}}$ is the input feature map, $\mathbf {A}\in {\mathbb {R}^{N \times N}}$ is the dense affinity matrix, and $\mathbf {Z}\in {\mathbb {R}^{C \times N}}$ is the output feature map. $\mathbf {W_{{g}}, W_{{f}}, W_{{h}}}\in {\mathbb {R}^{\Bar {C}\times C}}$, and $\mathbf {W_{{v}}}\in {\mathbb {R}^{C \times \Bar {C}}}$ are the learned weight matrices, which are implemented as 1×1convolutions. This mechanism reduces the channel number of *C* ¯ to be *C*/*k*, where *k*=1,2,4,8. The scaling factor *d* is used to solve the small gradient problem of softmax function according to [25] and $d=\frac {C}{2}$.

**Fig. 3:**
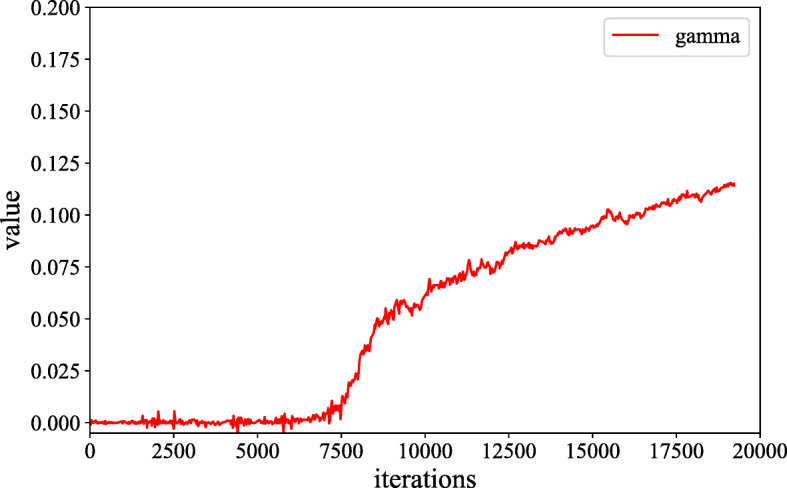
Illustration of how the parameter *γ* evolves during training

In addition, the output of the attention layer is multiplied with a scale parameter and add back the input feature map. Therefore, the final output is,

3 $$ \mathbf{Y}=\gamma\mathbf{Z}+\mathbf{X},  $$

where *γ* is a learnable scalar and it is initialized as 0. Introducing the learnable *γ* allows the network to first rely on the cues in the local neighborhood-since this is easier-and then gradually learn to assign more weight to the non-local evidence. As shown in Fig. 3, we find that in the training phase, the critical parameter *γ* slowly increases from the initial value zero with a small slope, then the increasing rate gradually becomes larger, and finally, the curve becomes stable.

#### Long-range attention

Long-range attention applies the self-attention on the subsets of positions that satisfy long spatial interval distances. As shown in Fig. 2, a permutation is first adopted on the input feature map **X** to compute **X**^*L*^=*Permute*(**X**). Then, **X**^*L*^ is divided into $\mathcal {P}$ parts and each part contains $\mathcal {Q}$ adjacent positions($N=\mathcal {P}\times \mathcal {Q}$). Here,

4 $$ \mathbf{X}^{L}=\left[{\mathbf{X}_{1}^{L}},{\mathbf{X}_{2}^{L}},...,{\mathbf{X}_{\mathcal{P}}^{L}}\right],  $$

5 $$ \mathbf{A}_{p}^{L}=softmax\left(\frac{\left(\mathbf{W}_{{f}} \mathbf{X}_{p}^{L}\right)^{\top}\left(\mathbf{W}_{{g}} \mathbf{X}_{p}^{L}\right)}{\sqrt{d}}\right),  $$

6 $$ \mathbf{Z}_{p}^{L}=\mathbf{W}_{{v}}\left[\left(\mathbf{W}_{{h}} \mathbf{X}_{p}^{L}\right)\mathbf{A}_{p}^{L}\right],  $$

7 $$  \mathbf{Z}^{L}=\left[{\mathbf{Z}_{1}^{L}},{\mathbf{Z}_{2}^{L}},...,{\mathbf{Z}_{\mathcal{P}}^{L}}\right],  $$

where $p=1,...,\mathcal {P}$, each $\mathbf {X}_{p}^{L} \in \mathbb {R}^{\mathcal {C} \times \mathcal {Q}}$ is a subset of $\mathbf {X}^{L}, \mathbf {A}_{p}^{L} \in \mathbb {R}^{\mathcal {Q} \times \mathcal {Q}}$ is the sparse affinity matrix based on all the positions from input feature map $\mathbf {X}_{p}^{L}$ and $\mathbf {Z}_{p}^{L} \in \mathbb {R}^{\mathcal {C} \times \mathcal {Q}}$ is the updated output feature map based on input feature map $\mathbf {X}_{p}^{L}$. All other parameters including **W**_*f*_,**W**_*g*_,**W**_*v*_,**W**_*h*_,*d* are the same as “Self-attention” section. Finally, all the $\mathbf {Z}_{p}^{L}$ is merged to acquire the output feature map **Z**^*L*^ in (7). From the equations above, we demonstrate the actual affinity matrix of long-range attention as below,

8 $$ \mathbf{A}^{L} = diag\left(\mathbf{A}_{1}^{L}, \mathbf{A}_{2}^{L},...,\mathbf{A}_{\mathcal{P}}^{L}\right),  $$

The equation shows that only the small affinity blocks in the diagonal are non-zero.

#### Short-range attention

Short-range attention applies the self-attention on the subsets of positions that satisfy short spatial interval distances. The decomposition principle is similar to the long-range attention mechanism.

#### Multi-level decomposition

The combination of long-range attention and short-range attention can effectively model global dependence. However, the computation of the small affinity matrix $\mathbf {A}_{p}^{L}$ from long-range attention is still not very efficient. We continue to decompose the sub-feature map $\mathbf {X}_{p}^{L}$. Here, we only perform two-level decomposition. As illustrated in Fig. 2, we first adopt a permutation on the input feature map **X** to compute **X**^*L*^ and divide **X**^*L*^ into $\mathcal {P}$ parts. Second, we apply a permutation on the input sub-feature map $\mathbf {X}_{p}^{L}$ to compute $\mathbf {X'}_{p}^{L}=Permute\left (\mathbf {X}_{p}^{L}\right) = \left [{\mathbf {X'}_{p1}^{L}},{\mathbf {X'}_{p2}^{L}},...,{\mathbf {X'}_{p\mathcal {P'}}^{L}}\right ],N'=\mathcal {P' \times Q'}$. Parameters here are similar to long-range attention. Then, we repeat the previous long-range attention and short-range attention steps in sequence, to calculate $\mathbf {Z}_{p}^{L_{L}}$ and $\mathbf {Z}_{p}^{L_{S}}$. After acquiring the updated output feature map based on the input sub-feature map $\mathbf {X}_{p}^{L}$, we merge all the $\mathbf {Z}_{p}^{L_{S}}$ to acquire the output feature map **Z**^*L*^. Finally, the output feature map **Z**^*S*^ can be obtained through performing short-range attention on **Z**^*L*^ directly.

#### Complexity of attention-aware layer

Given the input feature map of size *H*×*W*×*C*, we analyze the computation cost of the self-attention [33], interlaced sparse self-attention [30] and our proposed method.

The complexity of self-attention is

9 $$ \mathcal{O}\left(4HWC^{2}/k+2(HW)^{2}C/k\right),  $$

the complexity of interlaced sparse self-attention is

10 $$ \mathcal{O}\left(\frac{4HWC^{2}}{k}+\frac{\frac{3}{2}(HW)^{2}C}{k}\left(\frac{1}{\mathcal{P}_{h}\mathcal{P}_{w}}+\frac{1}{\mathcal{Q}_{h}\mathcal{Q}_{w}}\right)\right),  $$

and the complexity of our proposed method is

11 $$ \mathcal{O}\left(\frac{12HWC^{2}}{k}+\frac{2(HW)^{2}C}{k}\left(\frac{1}{\mathcal{P}_{h}\mathcal{P}_{w}}\left(\frac{1}{\mathcal{P'}_{h}\mathcal{P'}_{w}}+\frac{1}{\mathcal{Q'}_{h}\mathcal{Q'}_{w}}\right)+\frac{1}{\mathcal{Q}_{h}\mathcal{Q}_{w}}\right)\right)  $$

Where we divide the height dimension into $\mathcal {P}_{h}$ parts and the width dimension into $\mathcal {P}_{w}$ parts in long-range attention and $\mathcal {Q}_{h}$ and $\mathcal {Q}_{w}$ in short-range attention in the first level. In the second level, we divide the height $\mathcal {Q}_{h}$ and width $\mathcal {Q}_{w}$ again as the first level. Here, $H=\mathcal {P}_{h}\mathcal {Q}_{h}, W=\mathcal {P}_{w}\mathcal {Q}_{w}, \mathcal {Q}_{h}=\mathcal {P'}_{h}\mathcal {Q'}_{h}, \mathcal {Q}_{w}=\mathcal {P'}_{w}\mathcal {Q'}_{w}$. The complexity of interlaced sparse self-attention [30] can be minimized to $\mathcal {O}(4HWC^{2}/k+3(HW)^{\frac {3}{2}}C/k)$ when $\mathcal {P}_{h}\mathcal {P}_{w}=(HW)^{\frac {1}{2}}$. And the complexity of our method can be minimized to $\mathcal {O}(12HWC^{2}/k+6(HW)^{\frac {4}{3}}C/k)$ when $\mathcal {P}_{h}\mathcal {P}_{w}=(HW)^{\frac {1}{3}}$. It can be seen that our method has significantly lower computational complexity in processing high-resolution images than the first method.

### Loss function

EM images are quite different from natural images. These images have the characteristics of abundant noise and varying degrees of blur, which determine that general loss functions are not suitable for consecutive EM image interpolation. The loss function for training our network is a combination of *style balance loss*$\mathcal {L}_{bs}$, *feature reconstruction loss*$\mathcal {L}_{f}$, and *pixel-wise loss*$\mathcal {L}_{1}$. In all our experiments, *ϕ* is the 16-layer VGG network pretrained on ImageNet [34]. Specifically, we define the total loss as

12 $$ \mathcal{L}_{total}= \alpha_{1}\mathcal{L}_{bs} + \alpha_{2}\mathcal{L}_{f} + \alpha_{3}\mathcal{L}_{1}  $$

where the scalar *α*_1_,*α*_2_,*α*_3_ are the trade off weight, and the constant *α*_1_ is 10^6^, the constants *α*_2_=*α*_3_=1.

The proposed style balance loss aims at strengthening the supervision of the style of the middle frame, adopting the style of the front and back frames. The style reconstruction loss [35, 36] only ensures the style consistency between the generated intermediate frame and ground truth, ignoring the difference in style of consecutive EM images. Affected by the complex imaging environment of scanning electron microscopy, there are certain differences in the style of three consecutive EM images, such as blur, noise, brightness, and contrast. Considering that only frame *t*−1 and frame *t*+1 are input in the testing phase, we hope that the intermediate frames *t* generated by the model in the testing phase can take the styles of frame *t*−1 and frame *t*+1 into account and generate the intermediate frame *t*^′^ with natural style transitions. For this reason, we introduce style balance loss into the training phase to achieve a better balance between style transition and the style of ground truth. Here, we define the *Gram matrix* to be the *C*_*j*_×*C*_*j*_*matrix* and *ϕ*_*j*_(*x*) is the output of *j*th activation layer in vgg16 for the input *x*, the elements in *Gram matrix* are given by

13 $$ G_{j}^{\phi}(x)_{c,c'} = \frac{1}{C_{j}H_{j}W_{j}}\sum_{h=1}^{H_{j}}\sum_{w=1}^{W_{j}}\phi_{j}(x)_{h,w,c}\phi_{j}(x)_{h,w,c'},  $$

The *style reconstruction loss* is defined as below

14 $$ \mathcal{L}_{style} = \sum_{j=1}^{4}\left\|G_{j}^{\phi}(\hat{\mathbf{I}}_{t})-G_{j}^{\phi}(\mathbf{I}_{t}^{gt})\right\|_{2}^{2},  $$

The *style balance loss* is defined as

15 $$ \mathcal{L}_{bs} = \sum_{j=0}^{2}\beta_{j}sign\left(\mathcal{L}_{style_{1'j}}-\mathcal{L}_{style_{1j}}\right)\mathcal{L}_{style_{1'j}},  $$

where scalar *β*_0_,*β*_1_,*β*_2_ are the trade off weights and empirically set to 0.1,1,0.1 in turn. While making the style of the generated intermediate frame close to the ground truth, it also ensures the supervision of the frame 0 and frame 2 on the style of the generated intermediate frame 1^′^. $\mathcal {L}_{style_{1'j}}$ denotes *style reconstruction loss* between intermediate frame 1^′^ and input frame *j*, $\mathcal {L}_{style_{1j}}$ denotes *style reconstruction loss* between ground truth 1 and input frame *j*. If the frame 1 has a very different style from the frame 2, the value of the $\mathcal {L}_{style_{12}}$ is very large. When the style of frame 1^′^ tends to frame 1 and $sign\left (\mathcal {L}_{style_{1'2}}-\mathcal {L}_{style_{12}}\right)=1$, the third term in balanced loss is large and positive, and the network makes the style of frame 1^′^ approach frame 2 in a large slope; when the style of frame 1^′^ tends to frame 1 and $sign\left (\mathcal {L}_{style_{1'2}}-\mathcal {L}_{style_{12}}\right)=-1$, the third term is very large in absolute value and negative, and the network makes the style of frame 1^′^ move away from frame 2 in a large slope; If the frame 1 has a similar style to the frame 2, the value of $\mathcal {L}_{style_{1'2}}$ is very small. When the style of frame 1^′^ tends to frame 1, the third term in balance loss is close to zero and can be ignored; So either frame 0.

Feature reconstruction loss aims at achieving more realistic results, we adopt the *feature reconstruction loss* [35] to encourage the synthesized image $\hat {\mathbf {I}}_{t}$ and the ground-truth $\mathbf {I}_{t}^{gt}$ to have similar feature representations, defined as:

16 $$ \mathcal{L}_{f} = \sum_{j=1}^{4}\frac{1}{C_{j}H_{j}W_{j}}\left\|\phi_{j}(\hat{\mathbf{I}}_{t})-\phi_{j}\left(\mathbf{I}_{t}^{gt}\right)\right\|_{2}^{2},  $$

where *C*_*j*_×*H*_*j*_×*W*_*j*_ is the shape of output feature map *ϕ*_*j*_(*x*).

Pixel-wise loss aims at reducing the pixel-wise divergence between intermediate frames and ground truth. Here, we select Charbonnier loss [37] to resist outliers. The Charbonnier loss can be presented as:

17 $$ \mathcal{L}_{1} = \sum_{x}\rho\left(\hat{\mathbf{I}}_{t}(x)-\mathbf{I}_{t}^{gt}(x)\right),  $$

where $\hat {\mathbf {I}}_{t}(x)$ is the synthesized frame, $\mathbf {I}_{t}^{gt}(x)$ is the ground-truth frame, $\rho (x)=\sqrt {x^{2}+{\epsilon }^{2}}$ is the Charbonnier penalty function, and the constant *ε* is 10^−6^.

### Parameters in SSAN

The proposed models are optimized using the Adam [38] with the *β*_1_ of 0.9 and *β*_2_ of 0.999. We set the batch size to 3 with synchronized batch normalization. The initial learning rates of the proposed network are set to 10^−3^. We train the entire model for 30 epochs and then reduce the learning rate by a factor of 0.1 and fine-tune the entire model for another 20 epochs. Training requires approximately three days to converge on one Tesla K80 GPU. The whole SSAN framework is implemented using PyTorch.

For data augmentation, we randomly flipped the cropped patches horizontally or vertically and randomly swap their temporal order, for all datasets. All input images are randomly cropped to 512×512.

## Results

In this section, we first conduct an ablation study to analyze the contribution of the proposed loss function, feature extractor, and attention-aware layer. Then, we analyze the advantages of the proposed approach. Finally, we compare the proposed model with state-of-the-art algorithms on different EM datasets. The average Interpolation Error (IE), Peak Signal to Noise Ratio (PSNR), structural similarity (SSIM), graphics memory (Memory), Floating point number Operations Per Second (FLOPs), Module parameters (Params), run time (RunTime), Dice score (Dice) [39] and F1 score (F1) are computed for comparisons. Lower IEs, Memory, FLOPs and RunTime indicate better performance.

### Loss function analysis

**Fig. 4:**
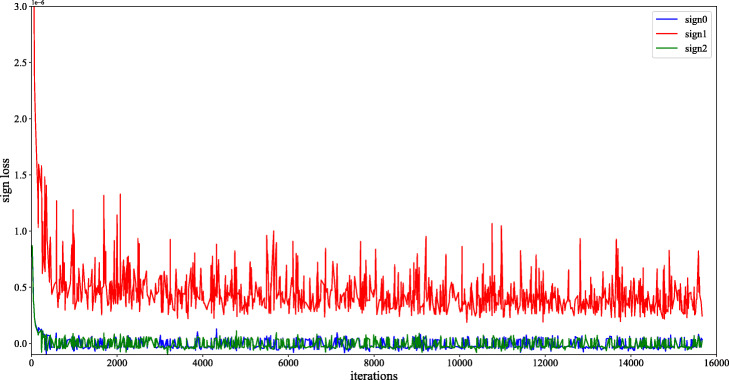
Illustration of how the three terms in the style balanced loss evolves during training. Here, *sign*0,*sign*1,*sign*2 denote $sign\left (\mathcal {L}_{style_{1'0}}-\mathcal {L}_{style_{10}}\right)\mathcal {L}_{style_{1'0}}, sign\left (\mathcal {L}_{style_{1'1}}-\mathcal {L}_{style_{11}}\right)\mathcal {L}_{style_{1'1}}$ and $sign\left (\mathcal {L}_{style_{1'2}}-\mathcal {L}_{style_{12}}\right)\mathcal {L}_{style_{1'2}}$, respectively

As shown in Fig. 4, We find that the three sub-items of the balance loss all tend to gradually approach zero from a larger value. The difference is that the first term and the third term gradually decrease from a small positive value to approach zero, the second term gradually decreases from a large positive value to approach zero, and the first and third terms are smaller than the second one and fluctuate around zero. The trend of the sub-items reflects in the figure above matches the style balance loss we have proposed.

**Fig. 5:**
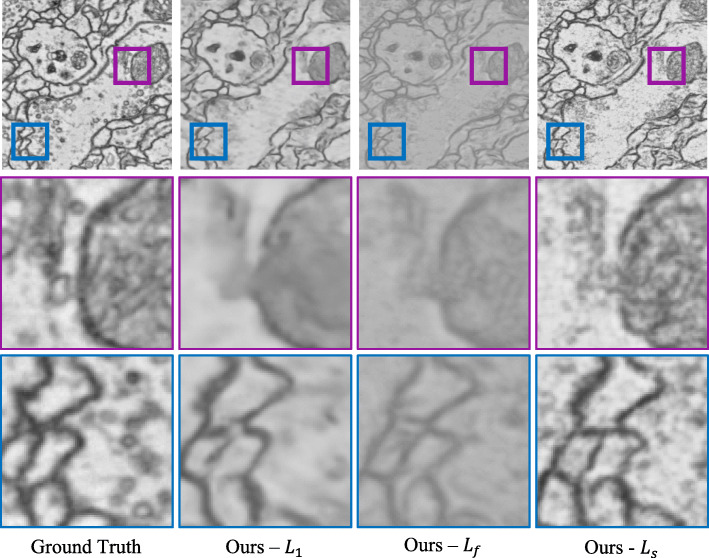
The results of the proposed model adopting different loss functions. From left to right: input frame 1, Ground Truth, result of $\mathcal {L}_{1}$, result of $\mathcal {L}_{f}$, result of $\mathcal {L}_{s}$, input frame 2

The proposed method incorporates three types of loss functions: pixel-wise loss $\mathcal {L}_{1}$, feature reconstruction loss $\mathcal {L}_{f}$ and style reconstruction loss $\mathcal {L}_{s}$. To indicate their respective effects, three different loss functions are adopted to train the proposed network. The first one only applies $\mathcal {L}_{1}$ loss and represents this network as “ *L*_1_”. The second one applies both $\mathcal {L}_{1}$ loss and $\mathcal {L}_{f}$ loss in a linear combination and represents this network as “ *L*_*f*_”. The third one applies $\mathcal {L}_{1}$ loss, $\mathcal {L}_{f}$ loss, and $\mathcal {L}_{bs}$ loss in a linear combination and represents this network as “ *L*_*s*_”. As shown in Fig. 5, the last “ *L*_*s*_” leads to the best visual quality and rich texture information. Results generated by “ *L*_*s*_” are visually pleasing with more high-frequency details. Despite slight deviation from the ground truth positions, results generated by “ *L*_*s*_” are consistent with biological tissue continuity. Also, the style of the images is almost the same as the ground truth. As a result, the proposed network adopts this scheme as the loss function.

### Model analysis

In this subsection, we analyze the contribution of the two key components in the proposed model: the siamese residual dense network (SRDN) and the attention-aware layer (AAL).

#### Siamese residual dense network

To validate the effectiveness of the SRDN, we compare it with other famous feature extractors, including the U-Net, the siamese U-Net (SU-Net), and the residual dense network (RDN) on the *cremi*_*triplet* datasets and *mouse*_*triplet* dataset. As shown in Table 1, the proposed SRDN feature extractor outperforms previous state-of-the-art feature extractors, achieving almost the best performance on PSNR, SSIM, and IE. Specifically, we demonstrate that the siamese structure, especially the siamese structure of RDN, leads to a substantial improvement on the *cremi*_*triplet* A and *mouse*_*triplet* in terms of PSNR and IE. We also find that RDN without siamese structure performs worse than U-Net, but under the siamese structure, the performance of RDN is significantly improved, which is better than U-net and SU-Net. We also notice the phenomenon that the Siamese structure does not help when applied to U-Net on the *cremi*_*tripletC* dataset. Compared with *cremi*_*tripletA* and *cremi*_*tripletB*, the deformation between three consecutive slices in the *cremi*_*tripletC* dataset is more complicated and changes drastically, which puts higher requirements on the network depth. The depth of the U-Net backbone is relatively shallow, and the Siamese structure would introduce bi-direction temporal information. However, pooling operation in U-Net leads to more information loss on the bi-direction deformation information than the single-direction, which accounts for the worse results on *cremi*_*tripletC* when applied Siamese structure to U-Net.

**Table 1:** Analysis on hierarchical features

Extractor	cremi_triplet A		cremi_triplet B		cremi_triplet C			mouse_triplet		
	PSNR	SSIM	PSNR	SSIM	PSNR	SSIM	IE	PSNR	SSIM	IE
U-Net [40]	18.10	0.4295	16.75	0.3549	16.26	0.3542	28.82	14.59	0.2219	36.55
SU-Net	18.12	0.4352	16.71	0.3417	16.18	0.3280	29.71	14.75	0.2089	35.99
RDN [32]	17.73	**0.4448**	16.59	0.3521	16.44	0.3451	28.75	14.85	0.2195	35.53
SRDN(ours)	**18.26**	0.4374	**16.79**	**0.3712**	**16.46**	**0.3575**	**28.38**	**15.04**	**0.2156**	**34.81**

We compared the actual effects of different feature extractors on the cremi_triplet datasets and mouse_triplet dataset. Lower IEs indicates better performance

#### Attention-aware layer

We demonstrate the superiority of the proposed AAL from two aspects: computational complexity and model effects. For the complexity of the attention perception module, all the numbers are tested on a single P40 GPU with cuda10.2, and the input feature map resolution is 1×64×512×512. As shown in Table 2, it can be seen that the proposed AAL only uses 23.8*%* GPU memory and 12.8*%* FLOPs compared with the SSA. Besides, the running time of our method is 275 ms, which is 52 ms faster than SSA. The results sufficiently demonstrate that the computation and memory complexity of proposed method are substantially lower than other self-attention methods. To validate the effectiveness of the proposed attention-aware layer, the feature extractor adopts a siamese residual dense network. After the feature extractor, we append the classic kernel estimation layer, the state-of-the-art interlaced sparse self-attention layer, and the proposed attention-aware layer, respectively. For the implementation of the self-attention, we directly utilize the open-source code [33]. As shown in Table 3, the proposed AAL shows an improvement on the *cremi*_*triplet* and *mouse*_*triplet* datasets, against both KEL and SSA. Especially on the *mouse*_*triplet*, AAL outperforms SSA with a 0.18dB gain in terms of PSNR. Meanwhile, the interpolation error (IE) is 0.6 lower than SSA.

**Table 2:** GPU memory/FLOPs/Params/RunTime comparison on kernel estimation layer (KEL), self-attention (SA), interlaced sparse self-attention layer (SSA) and the proposed approach

	Memory(GB)	FLOPs(G)	Params(M)	RunTime(ms)
KEL [9]	-	138.4	-	6160
SA [27]	256.0	6599	**0.0052**	-
SSA [30]	9.775	81.24	0.0104	327
AAL(Ours)	**2.331**	**10.42**	0.0156	**275**

All the numbers are tested on a single P40 GPU with CUDA10.2 and an input feature map of 1×64×512×512 during inference stage. Lower Memory, FLOPs and RunTime indicate better performance

**Table 3:** Effects on attention-aware layer (AAL)

Synthesis	cremi_triplet A		cremi_triplet B		cremi_triplet C			mouse_triplet		
	PSNR	SSIM	PSNR	SSIM	PSNR	SSIM	IE	PSNR	SSIM	IE
KEL [9]	17.59	0.4354	15.88	0.3415	16.08	0.3506	30.82	13.46	0.1867	42.62
SSA [30]	18.12	0.4292	16.41	0.3425	16.33	0.3357	28.91	14.86	**0.2261**	35.41
AAL(Ours)	**18.26**	**0.4374**	**16.79**	**0.3712**	**16.46**	**0.3575**	**28.38**	**15.04**	0.2156	**34.81**

Compared with kernel estimation layer (KEL) and interlaced sparse self-attention layer (SSA), the proposed attention-aware layer (AAL) presents a significant improvement on both the cremi_triplet and mouse_triplet datasets. Lower IEs indicates better performance

### Analysis of the proposed approach

**Fig. 6:**
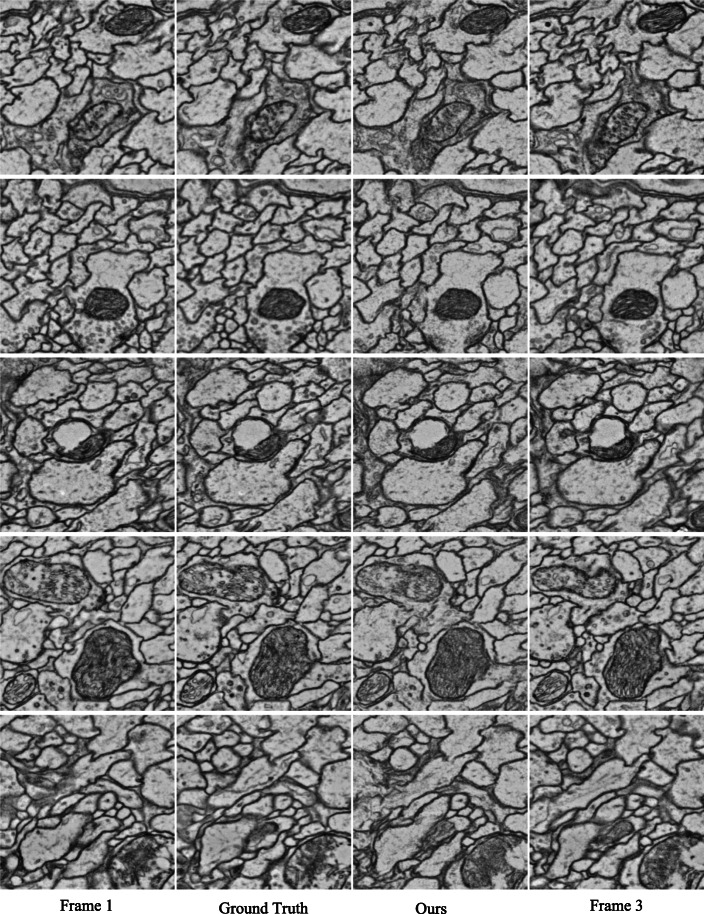
Results of our proposed method on cremi_triplet B dataset. From top to bottom: input frame 1, ground truth, generated indemediate frame and input frame 3

As shown in Fig. 6, qualitative visualization results on cremi_triplet B can demonstrate the superiority of the proposed method. The intermediate EM images generated by our proposed method are almost the same as ground truth, in terms of image style, biological tissue continuity, and content texture. The proposed attention perception layer can synthesize each pixel of the intermediate frame from the global domain. Thus, the proposed approach is robust against large deformations, drifts, and noise. We can observe that in the case of many discontinuous pixels in the input frame and the ground truth, this approach can produce ideal results.

### Comparisons with state-of-the-arts

**Fig. 7:**
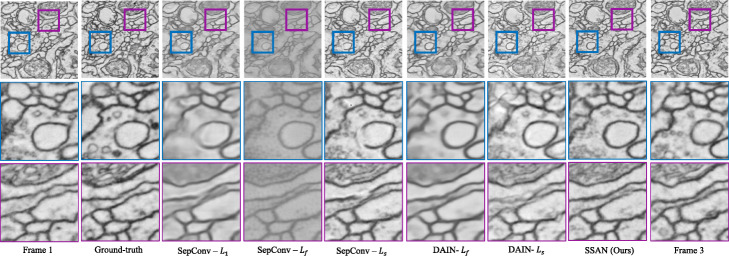
Visual comparisons on the cremi_triplet B. To demonstrate the superiority of the proposed method, we visualize the results of the comparison methods using different loss functions. The proposed method not only has almost the same style as the true value, but also obtains a clear outline and a more realistic content texture

We conducted quantitative and qualitative experiments on the proposed approach and the baseline to prove that the proposed method is superior to the baseline. For quantitative experiments, we adjust the loss function of both the proposed method and the baseline to the style balance loss introduced in this paper and conduct quantitative experiments under the same experimental environment. In Table 4, we provide quantitative performances on the *cremi*_*triplet* A, *cremi*_*triplet* B, *cremi*_*triplet* C, and *mouse*_*triplet* dataset. The proposed approach performs favorably against all the compared methods for all the datasets, especially on the mouse_triplet dataset with a 2.48dB gain over SepConv-L _*s*_ [9] in terms of PSNR. For qualitative experiments, all methods are conducted under the same experimental environment and apply different loss functions to intuitively demonstrate the effect of varying loss functions on EM images. We evaluate the proposed SSAN against the following CNN-based frame interpolation methods: SepConv-L_1_ [9], SepConv-L _*f*_ [9], SepConv-L _*s*_ [9], DAIN-L_1_ [12] and DAIN-L _*s*_ [12], in terms of PSNR, SSIM and IE. As shown in Fig. 7, the DAIN-L_1_ [12] and DAIN-L _*s*_ [12] cannot handle the large deformation well and thus produce ghosting and broken results. Moreover, we can see the lack of some edges from the enlarged image. It confirms that flow-based methods perform poorly on EM images. The SepConv-L_1_ [9] and SepConv-L _*f*_ [9] methods generate blurred results on membrane structure and mitochondria. The result generated by SepConv-L _*s*_ [9] also lacks critical edge information, and there are black noise and white areas inconsistent with the continuity of biological tissue, especially around mitochondria. In contrast, the proposed method handles large deformation well and generates clearer results with complete contour.

**Table 4:** Quantitative comparisons on cremi_triplet and mouse_triplet

Methods	cremi_triplet A		cremi_triplet B		cremi_triplet C			mouse_triplet		
	PSNR	SSIM	PSNR	SSIM	PSNR	SSIM	IE	PSNR	SSIM	IE
SepConv-L _*s*_ [9]	17.52	0.4095	16.32	0.3522	16.07	0.3454	29.82	12.56	0.1573	48.03
DAIN-L _*s*_ [12]	16.78	0.4264	15.67	0.3460	15.24	0.3210	33.59	13.06	0.1973	44.26
SSAN(Ours)	**18.26**	**0.4374**	**16.79**	**0.3712**	**16.46**	**0.3575**	**28.38**	**15.04**	**0.2156**	**34.81**

The proposed SSAN algorithm significantly surpasses other methods in terms of PSNR, SSIM and IE

### Segmentation performance comparisons with state-of-the-arts

**Fig. 8:**
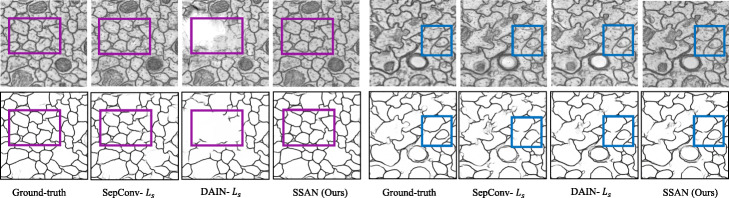
Visual comparison of segmentation results on the cremi_triplet A. To demonstrate the superiority of the proposed method, we visualize the segmentation performance of the comparison methods

Table 5 shows the segmentation accuracy attained by each method. In all cases, our proposed SSAN algorithm performs best than the other two methods, both in Dice score and F1 score. Because it uses not only the SRDN to avoid the information loss caused by the pooling operation and retain the temporal information, but also the sparse self-attention to synthesize each pixel considering the long-range dependence. Thus, the inter-slice generated by SSAN can produce clear and accurate membrane boundaries and fewer artifacts despite large deformation, noise, and blur. From Fig. 8, we visualize the membrane segmentation results of intermediate images generated by different methods using the same L _*s*_ loss. It can be seen that the intermediate frame generated by the proposed method has fewer artifacts and the membrane boundary is more complete. The flow-based method is unstable on the EM images, and even produces severe white spots.

**Table 5:** Segmentation performance of different methods in terms of the Dice score and F1 score, evaluated on cremi_triplet

Methods	cremi_triplet A		cremi_triplet B		cremi_triplet C	
	Dice	F1	Dice	F1	Dice	F1
SepConv-L _*s*_ [9]	0.547	0.546	0.425	0.426	0.458	0.458
DAIN-L _*s*_ [12]	0.560	0.563	0.340	0.342	0.384	0.388
SSAN(Ours)	**0.602**	**0.602**	**0.435**	**0.435**	**0.465**	**0.467**

We report the mean metrics of the membrane boundary on the ground truth and intermediate image synthesized by different methods. The proposed SSAN algorithm significantly surpasses other methods in these evaluation metrics

## Discussion

In this work, we consider the sparse self-attention mechanism and discuss how to introduce this self-attention into consecutive EM image interpolation tasks. On EM images with large deformation, drift, and abundant noise, each pixel of the intermediate frame is aggregated from all positions in the input frame using a self-attention mechanism. Specifically, we highlight three aspects: feature extraction module, attention perception mechanism, and style balance strategy. We found that U-Net’s pooling can damage content information, and the residual dense blocks commonly adopted in super-resolution preserve the integrity of content information. The Siamese structure in the feature extractor enables the network to extract the temporal information among the input frames. We empirically observe that a two-level sparse strategy decreases the computation and memory complexity substantially while performing better in synthesizing pixels from the input frames’ global domain. Given the input feature map of size *H*×*W*×*C*, the complexity of interlaced sparse self-attention [30] can be minimized to $\mathcal {O}(4HWC^{2}/k+3(HW)^{\frac {3}{2}}C/k)$, and the complexity of the proposed method can be minimized to $\mathcal {O}(12HWC^{2}/k+6(HW)^{\frac {4}{3}}C/k)$. Thus, our method has a significantly lower computational complexity than the first one while entering high resolution feature maps. After obtaining warped frames, we found that only simple averaging does not give good results. The sigmoid function is a good alternative that generates a weight mask for element-wise linearly fusing two warped frames to synthesize the interpolated frame. We observe that selecting a suitable loss function for training models on EM images is much more complicated than natural images, especially to be robust against large deformations, drifts, and noise. We conducted a combined experiment on style loss, perceptual loss, and pixel loss, and found that the ratio 10^6^:1:1 produced the most realistic results. We further proposed an adaptive style balance loss to ensure a natural transition of three consecutive frame styles. Finally, our proposed approach performs better than other methods on EM images, produced by ssTEM and ATUM.

In the future, one option is to sparse from the self-attention mechanism to reduce computation and memory consumption further. Another option is to optimize the loss further and propose a novel loss that is more suitable for the task of EM image interpolation. The last improvement direction is to design a sparse global domain kernel estimation method.

## Conclusion

In this paper, we propose a novel attention-aware consecutive EM image interpolation algorithm that combines motion estimation and frame synthesis into a single process by adopting the AAL. The proposed AAL implicitly detects the large deformations using the self-attention information and synthesize each pixel by effectively establishing long-range dependencies from input frames. The AAL entirely replaces the traditional kernel estimation convolution method with low memory and computational consumption. We also exploit the SRDN as the feature extractor to learn hierarchical features and reduce the parameters. Furthermore, the proposed adaptive style balance loss takes the style information of input EM images into consideration, generating more realistic results. Our SSAN performs more favorably on EM images than flow-based methods due to integrating flow estimation and pixel synthesis through the attention-aware mechanism. The experiments on ssTEM and ATUM images show that the proposed approach compares favorably to state-of-the-art interpolation methods, both quantitatively and qualitatively, and generates high-quality frame synthesis results.

## Data Availability

The data and source code in this paper are available online.

## Footnotes

1 https://cremi.org/
